# Comparison of open liver resection and RFA for the treatment of solitary 3–5-cmhepatocellular carcinoma: a retrospective study

**DOI:** 10.1186/s12893-019-0663-9

**Published:** 2019-12-16

**Authors:** Lei Jianyong, Yan Lunan, Li Dajiang, Wang Wentao, Liver Surgery Group

**Affiliations:** 1 Thyroid and Parathyroid Surgery Center, West China Hospital of Sichuan University, Chengdu, 610041 China; 2 Liver Surgery, West China Hospital of Sichuan University, Chengdu, 610041 China; 3 The Medical Department, West China Hospital of Sichuan University, Chengdu, 610041 China

**Keywords:** Hepatocellular carcinoma, Liver, Resection, Radiofrequency ablation

## Abstract

**Background:**

The goal of this study was to compare the postoperative results of liver resection and radiofrequency ablation (RFA) for the treatment of small hepatocellular carcinoma (HCC) (3–5 cm).

**Patients and methods:**

We retrospectively collected 122 cases of small solitary HCC treated at our center from Jan 2011 to Dec 2015, with diameters in the range of 3–5 cm. According to the treatment program received at our center, the patients were divided into liver resection (72 patients) and RFA (50 patients) groups.

**Result:**

In comparison with the RFA group, the resection group had a longer operative time, greater intraoperative blood loss (*P* < 0.01), more hepatic inflow occlusion, and a longer postoperative hospital stay (*P* < 0.01). The 1-, 3-, and 5-year expected overall survival rates and tumor-free survival rates were comparable between the two groups. Cox regression analysis showed that neither resection nor RFA was a significant risk factor for overall or tumor-free survival in HCC.

**Conclusions:**

For solitary HCC of 3–5 cm in diameter, RFA can achieve better in-hospital clinical results and similar long-term outcomes than resection and can be considered for wide application, especially for central-location cases.

## Background

Hepatocellular carcinoma (HCC) ranks fifth in the global incidence of malignant tumors, and HCC-related mortality ranks third [1]. The burden imposed by the diagnosis of liver cancer is particularly prominent in China [2]. For the treatment of early-stage liver cancer, commonly accepted radical treatments include liver transplant (LT), liver resection, and radiofrequency ablation (RFA). LT not only removes the lesion but also removes the substrate for growth of the tumor, and it is generally considered the most effective method [3]. However, liver resection and RFA are the main treatments for early HCC. Comparison of safety and effectiveness between liver resection and RFA has been the subject of much research interest for the past several years [4].

Although a unified understanding has not been reached, analysis of effectiveness has generally indicated that long-term survival and tumor recurrence are similar between these methods for small liver tumors with a diameter ≤ 3 cm [5]; for liver tumors with a diameter > 5 cm, it is currently believed that RFA cannot achieve the effect of radical treatment [6]. However, for liver tumors with diameters of 3–5 cm, there is still considerable controversy regarding the effect of RFA [7–9]. Additionally, most studies comparing these two methods have focused on the treatment or control of the tumor, whereas the safety of the two treatment methods has been ignored. Nonetheless, safety remains an issue that must be considered when selecting treatment. Therefore, this study was performed to comprehensively examine the efficacy and safety of liver cancer (diameter 3–5 cm) treatment with resection and RFA using data collected at our center.

## Methods

### Patients

This retrospective study was performed after approval from the Ethics Committee of our hospital. Written informed consent was obtained from all patients for publication of data as well as any accompanying images and videos. We retrospectively collected all liver cancer patients admitted to our hospital from Jan 2011 to Dec 2015; the cases were screened according to inclusion and exclusion criteria (shown in Table 1). Ultimately, we collected 122 patients for group analysis.

**Table 1:** Main inclusion/exclusion criteria of the study

Inclusion criteria	
Single tumor	
3 cm ≤ diameter of tumor< 5 cm	
ECOG score 0–1	
Liver function of grade Child A or B	
Receiving the first treatment in our center: liver resection or RFA	
All cases were accomplished with laparotomy	
Able to receive the complete postoperative regular follow-up visit or inspection	
Exclusion criteria	
Tumor thrombus in major vessels	
Multiple tumors, or distant metastasis	
The heart and lung function of the patients cannot tolerate surgical treatment	
Cannot take the surgery due to other diseases	
Cases with preoperative intervention of HIFU knife on the lesion or other preoperative relevant downgrade treatment	
Liver function at level C (patients achieving levels A or B after liver-protection treatment can be incorporated)	
Cases with postoperative diagnosis of biliary carcinoma or other non-liver-cell liver cancer	
Cases that received other treatment means	
Cases without follow-up after the surgery	
Other treatment cases that adopted intravenous chemotherapy or took sorafenib after the surgery	
Other surgical contraindications, such as coagulation disorders	

We divided the 122 patients into a liver resection group and an RFA group according to the treatment strategy applied, with the liver resection group including 72 patients and the RFA group 50 patients. We compared the following intraoperative data for the two groups: surgical time, blood loss due to hepatic portal occlusion, and transfusion rate. We also compared relevant postoperative data, and the Clavien evaluation system was employed to assess the occurrence of postoperative complications. We then compared 1-, 3-, and 5-year overall survival rates and tumor-free survival rates. The surgical procedures and follow-up protocol have been introduced in our previous publications [10–12].

### Statistical analysis

We used SPSS 17.0 (SPSS Inc. Chicago, IL, USA) for statistical analysis of the data. Patient baseline characteristics and other continuous variables were compared and calculated using the non-parametric Wilcoxon test because some of the measurements did not have normal distributions; the results are expressed as the mean ± SD. Categorical data, which are expressed as frequencies, were compared using the Chi-squared test or Fisher’s exact test, as appropriate. Ranked data were compared using the Mann-Whitney U test. Overall survival and tumor-free survival rates were obtained by Kaplan-Meier survival analysis, and differences in survival curves between the two groups were statistically compared by the log-rank test. Univariate analysis was performed to identify factors predicting overall and tumor-free survival. All variables with *P* < 0.05 were included in multivariate analysis using Cox regression to assess independent predictive factors. Two-sided *P* values < 0.05 were considered statistically significant. Ultimately, biological characteristics and common sense must be combined to determine clinical significance.

## Results

### Clinical and pathological characteristics

As shown in Table 2, we found no significant difference in demographic baseline characteristics among the patients in the two groups (*P* > 0.05). Furthermore, differences in preoperative liver function between the two groups was not statistically significant according to either the Child scoring system or the MELD scoring system.

**Table 2:** Comparison between baseline data and oncological features of patients in resection group and RFA group

	Liver resection group	RFA group	*P* value
	72	50	
Age	46.7 ± 10.3	45.8 ± 12.0	0.664
Gender (male/female)	61/11	42/8	0.914
Weight (Kg)	68.1 ± 9.5	66.8 ± 10.0	0.474
Height (cm)	166.9 ± 8.4	163.5 ± 8.4	0.057
BMI (kg/m^2^)	23.3 ± 2.4	23.8 ± 1.8	0.252
Ethnicity(Han//Tibet/Yi/Others)	63/4/2/3	45/2/2/1	0.665
Virological examination			
(B/C/negative)	67/1/4	46/4/0	0.899
HBV-DNA (negative/positive)	28/39	25/21	0.191
Child score (A/B/C)	47/25/0	37/13/0	0.308
MELD score	5.5 ± 2.2	5.4 ± 1.5	0.688
Ishak score	3.9 ± 1.5	4.1 ± 1.2	0.411
Tumor diameter (cm)	3.7 ± 0.5	3.8 ± 0.5	0.528
Preoperative AFP level (ng/ml)	3781.9 ± 14,105.9	4716.6 ± 14,813.6	0.725
Preoperative AFP (−/+/++/+++)	33/10/10/19	19/8/8/15	0.444
Degree of tumor differentiation			
(low/moderate/high)	15/27/30	16/19/15	0.116
Tumor location (edge/center)	53/19	12/38	0.000
Microvascular invasion (yes/no)	23/49	17/33	0.813

HBV DNA negative: < 1.0E+ 03 copies/ml, positive: ≥1.0E+ 03 copies/ml

Other ethnicities: Qiang and Mongolian

Preoperative AFP level: -: < 12 ng/ml; +: 12 ng/ml ≤ < 400 ng/mL; ++: 400 ng/ml < ≤1200 ng/mL; +++: ≥1200 ng/ml

Continuous variables compared and calculated by using non-parametric Wilcoxon tests, frequencies for categorical data, and compared by using the Chi-squared test or Fisher’s exact test if necessary, ranked data were compared by using Mann-Whitney U test

Regarding the comparison of cancer characteristics, the cases analyzed all involved single lesions. Although our results indicated that the tumor diameter for the RFA group was slightly larger than that for the resection group (3.8 vs. 3.7 cm), this difference was not statistically significant (*P* = 0.528). Regarding the position of the tumor in the liver, the tumor was located on the edge in 53 cases of the resection group (73.6%) but in only 12 cases (24.0%) for the RFA group (*P* < 0.01). This difference is mainly because the advantages and disadvantages of RFA and resection cause the surgeon to adopt the optimal treatment plan before or during surgery.

### Intraoperative and short-term outcomes

Table 3 presents the comparison of relevant intra- and postoperative data for the resection and RFA groups. The operative time of the resection group was significantly longer than that of the RFA group (4.0 vs. 2.7 h, *P* < 0.001). In addition, intraoperative blood loss was significantly greater in the resection group than in the RFA group (364.6 vs. 102.0 ml, *P* < 0.001), and hepatic portal occlusion occurred significantly more often in the resection group than in the RFA group (*P* < 0.001). We also found that the average hospital stay of the resection group was 6.3 days, which was significantly longer than the 4.7 days for the RFA group (*P* = 0.001).

**Table 3:** Comparison of relevant intra-operative data and post-operative short-term recovery situation between the two groups

	Resection group	RFA group	*P* value
	72	50	
Operation time (hours)	4.0 ± 1.2	2.7 ± 0.8	< 0.001
Intra-operative blood loss (ml)	364.6 ± 180.1	102.0 ± 105.9	< 0.001
Intra-operative transfusion (Yes/No)	9/63	3/47	0.238
Hepatic inflow occlusion (whole liver/half	9/36/27	2/4/44	< 0.001
liver/non-blocking)			
ICU care (Yes/No)	5/67	3/47	0.836
Total number of days in hospital	6.3 ± 2.2	4.7 ± 1.8	0.001
Total cost of hospitalization (RMB Yuan)	35,542 ± 2456.9	33,453.2 ± 1986.6	0.939

*ICU* Intensive care unit

Continuous variables compared and calculated by using non-parametric Wilcoxon tests, frequencies for categorical data, and compared by using the Chi-squared test or Fisher’s exact test if necessary,

We adopted the Clavien system to summarize and compare the occurrence of postoperative complications between the two groups (Table 4). In the resection group, 18 patients experienced complications, for a total occurrence rate of 25%; in the RFA group, there were 8 patients with complications, for a total occurrence rate of 16%. Although the occurrence rate of complications was higher in the resection group than in the RFA group, the difference was not statistically significant (*P* = 0.284). The occurrence rate of serious complications (≥level III) was 8.3% for the resection group and 4% for the RFA group, with no significant difference (*P* = 0.344). In the RFA group, there was one case of postoperative fever. Color ultrasonography and computed tomography (CT) revealed the formation of biloma with abscess, and we performed laparotomy abdominal inspection to conduct a partial liver resection.

**Table 4:** Comparison of post-operative complication occurrence for resection group and radiofrequency group (Clavien scoring system)

	Resection group	RFA group
	72	50
Clavien level I (without drugs, conservative treatment)	7 (9.7%)	4 (8%)
Incision fat liquefaction	2	1
Wound infection	1	2
Pleural effusion	2	1
Biliary fistula	2	0
Clavien level II (simple medicine treatment)	5 (6.9%)	2 (4%)
Wound infection	2	1
Postoperative pulmonary infection	1	1
Postoperative abdominal hemorrhage	2	0
Clavien level IIIa (therapeutic operation	1 (1.4%)	0 (0%)
under local anesthesia)		
Pleural effusion	1	0
Clavien level IIIb (operational treatment	1 (1.4%)	1 (2%)
under general anesthesia)		
Abdominal hemorrhage	1	0
Biloma	0	1
Clavien level IVa (single organ function failure)	2 (2.8%)	1 (2%)
Respiratory failure	1	0
Liver failure	1	1
Clavien level IVb (multiple organ failure)	1 (1.4%)	0 (0%)
Hepatorenal syndrome	1	0
Clavien level V (death)	1 (1.4%)	0 (0%)
Septic shock	1	0

### Long-term outcome

**Fig. 1:**
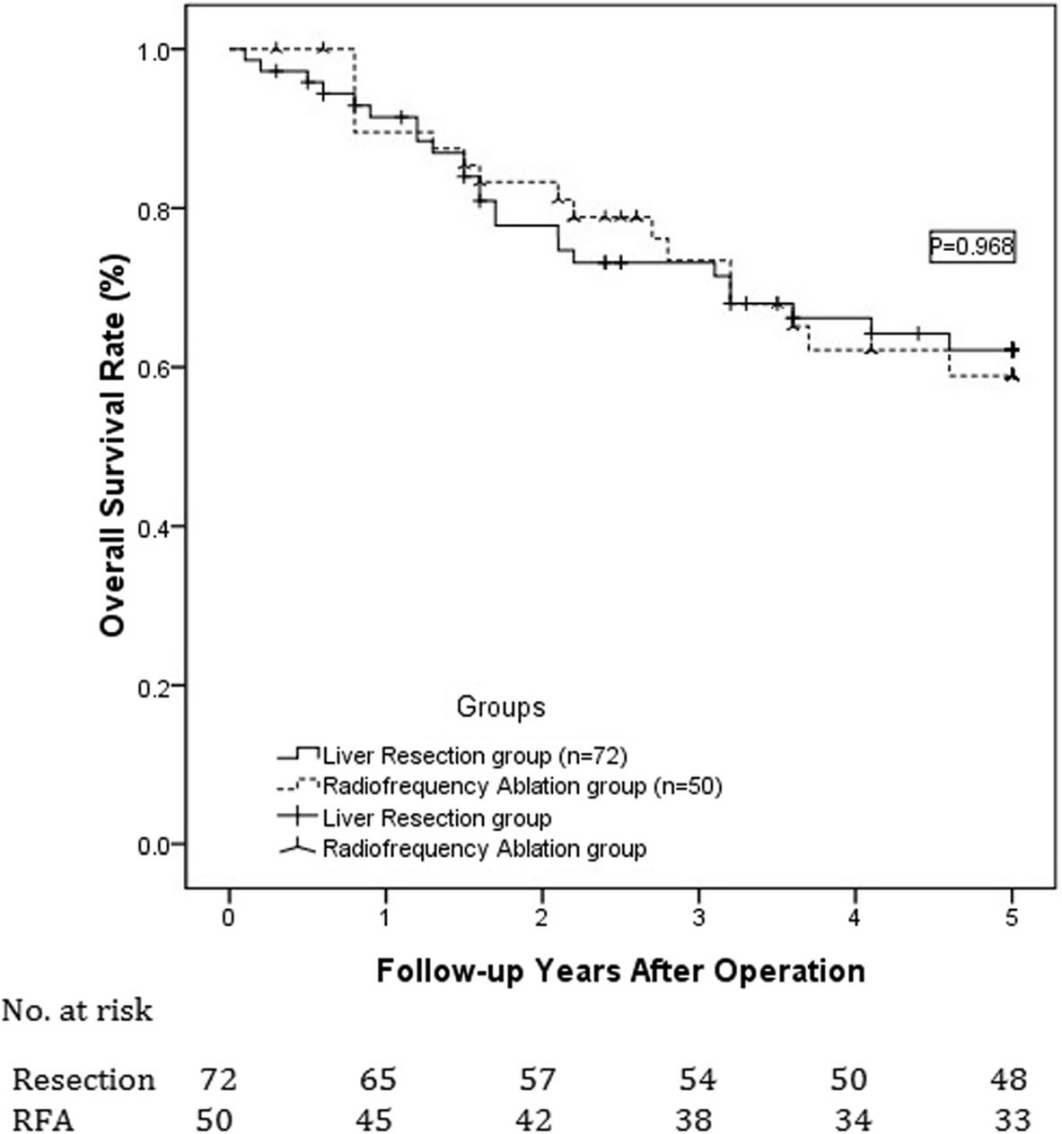
Comparison of 1-, 3-, and 5-year expected overall survival rates for the treatment of small liver cancer with diameters of 3–5 cm between abdominal RFA and resection groups; no significant differences were found. For the resection group, the values were 94.4, 77.8, and 70.8%, respectively, and for the radiofrequency group, the values were 90, 76, and 68%, respectively (*P* = 0.968)

**Fig. 2:**
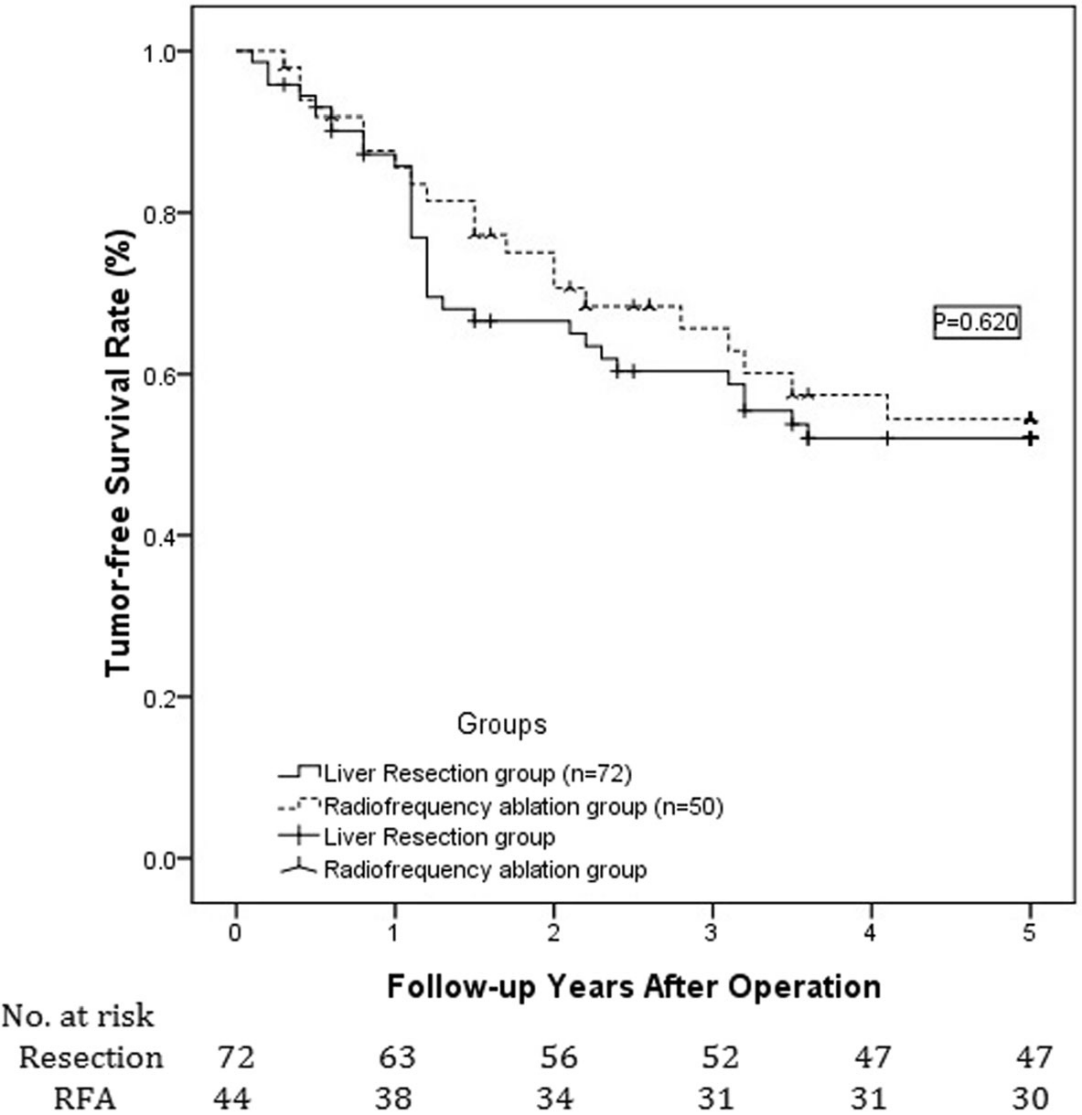
Comparison of 1-, 3-, and 5-year expected tumor-free survival rates for patients in resection and RFA groups with respect to small liver tumors with diameters of 3–5 cm. For the resection group, these values are 87.5, 62.5, and 55.6%, respectively, and for the RFA group, these values are 88, 68, and 60%, respectively (*P* = 0.620)

During the follow-up period, the 1-, 3-, and 5-year expected overall survival rates for the resection group were 94.4, 77.8, and 70.8% vs 90, 76, and 68% for the RFA group, as illustrated in Fig. 1. However, these differences were not statistically significant (*P* = 0.968). The 1-, 3-, and 5-year expected tumor-free survival rates were 87.5, 62.5, and 55.6% and 88, 68, and 60% in the resection and RFA groups, respectively. As shown in Fig. 2, the difference between the two groups was not statistically significant (*P* = 0.620). During the follow-up period, 41 patients died, and 52 experienced HCC recurrence. The most common recurrence or metastasis location of postoperative tumors was the liver (55%). Extrahepatic recurrence and metastasis occurred mostly in the lung (30%), followed by intra-abdominal metastasis (7.5%); bone metastasis was rare (5%), as was metastasis to other parts of the body (2.5%). The most common treatment for tumor recurrence was transcatheter arterial chemoembolization (TACE) (28 cases), followed by re-resection (11 cases) or RFA (21 cases); in contrast, LT (2 cases), high-intensity focused ultrasound (HIFU) knife (3 cases), and other treatment programs [including sorafenib (2 cases), chemotherapy (1 case), and radiotherapy (1 case)] were rare. The most common factor causing death during the postoperative follow-up period was tumor recurrence and metastasis (78%), followed by liver function failure (19.5%); other causes were rare (2.4%).

### Univariate and multivariate analyses

As shown in Table 5, univariate analysis identified the preoperative neutrophil-lymphocyte ratio (NLR) ≥ 4 (*P* < 0.001), AFP ≥400 ng/ml (*P* = 0.014), intra-operative blood loss≥400 ml (*P* = 0.029), poor histological grade (*P* < 0.029), central tumor location (*P* = 0.044), and microvascular invasion (*P* < 0.001) as being significant factors contributing to overall survival after surgery. Multivariate analysis of the five factors found to be significant in univariate analysis further identified NLR ≥ 4 (*P* = 0.020) and poor histological grade (P < 0.001) as significant contributors to overall survival. The hazard ratios (HRs) and 95% confidence intervals (CIs) for these factors are provided in Table 6.

**Table 5:** Univariate analyses contributing to overall survival and tumor-free survival rate after RFA or Resection

Variables	N (122)	Overall survival rate	Tumor-free survival rate
		*P* Value	*P* Value
Age ≥ 60 (yes/no)	19/103	0.398	0.961
Gender (M/F)	103/19	0.398	0.652
Race (Han/other)	108/14	0.170	0.083
BMI ≥ 26 (yes/no)	13/109	0.821	0.751
Causes of liver diseases (HBV/other)	113/9	0.150	0.419
Child Score (A/B)	84/38	0.255	0.389
Hemoglobin <120 g/L(yes/no)	44/78	0.480	0.766
Platelet <100*10^9/L(yes/no)	40/74	0.194	0.029
NLR ≥ 4 (yes/no)	62/60	<0.001	<0.001
AFP ≥400 ng/ml (yes/no)	52/70	0.014	0.059
Tumor diameter (3–4/4–5)	56/66	0.488	0.296
Radical therapy (RFA/ Resection)	50/72	0.940	0.629
Tumor location (center/edge)	57/65	0.044	0.358
Intra-operative blood loss≥400 ml	38/84	0.029	0.161
(yes/no)	44/45/33	<0.001	0.212
Histological grading (well/moderate/poor) Microvascular invasion (yes/no)	40/82	<0.001	<0.001

*NLR* Neutrophil-lymphocyte ratio, *AFP* Alpha-fetoprotein *BMI* Body mass index, *HBV* Hepatitis B virus, *M* Male; *F* Female

**Table 6:** Multivariate analyses contributing to overall survival and tumor-free survival rate

Variables	Hazard ratio	95% CI	*P*-value
Prognostic factors for overall survival			
NLR ≥ 4	1.453	1.072–2.287	0.020
AFP ≥400 ng/ml Intra-operative blood loss ≥ 400 ml	1.864 1.219	1.021–3.210 0.829–2.083	0.102 0.398
Histological grading			
Well			
Moderate	2.211	1.806–3.127	0.046
Poor	2.680	1.346–4.632	<0.001
Tumor located central	1.458	1.091–1.762	0.469
Microvascular invasion	2.209	1.210–3.290	0.016
Treatment modality (Resection/RFA)	1.542	1.105–3.026	0.895
Prognostic factors for tumor-free survival			
Platelet <100*10^9/L	1.782	1.142–2.891	0.142
NLR ≥ 4	1.374	1.201–2.347	<0.001
Microvascular invasion	1.618	1.082–2.289	0.015
Treatment modality (Resection/RFA)	1.762	1.052–2.217	0.651

*NLR* Neutrophil-lymphocyte ratio, *AFP* Alpha-fetoproteinCox regression was used in multivariate analysis

Table 5 shows that preoperative platelet count < 100*10^9^/L (*P* = 0.029), NLR ≥ 4, and microvascular invasion (P < 0.001) were significant factors contributing to lower long-term tumor-free survival. Multivariate analysis indicated NLR ≥ 4 and microvascular invasion as risk factors for tumor recurrence (Table 6).

## Discussion

In general, surgical resection is a more radical treatment approach than is RFA. However, due to the limitations of liver condition and function, postoperative complications also need to be taken into account. RFA surgery is a relatively safe treatment approach, but the stability and thoroughness of RFA for treating liver cancer are difficult to determine [13, 14]. Although evaluation of effectiveness between the two approaches is still being performed, in Western countries, especially in the United States, treatment guidelines for liver cancer consistently recommend surgical resection for early liver cancer in cases of adequate liver function and the absence of high venous pressure [15]. However, the guidelines also mention that due to the nearly 3% mortality rate after liver resection surgery, the use of other therapies to treat small liver tumors may be appropriate, with ablation treatment being preferred. At present, RFA is the relevant mature ablation treatment method, and there have been many studies on the effectiveness and reliability of RFA and surgical resection for the treatment of liver cancer. Despite the lack of a unified opinion, we believe that the effect of RFA depends on the maximum diameter of the tumor. Current studies mostly define RFA as the gold standard for tumors smaller than 2.3–3 cm. For a single tumor with a diameter smaller than 3 cm, RFA can achieve results similar to those of resection, and the safety of the patient is ensured [16–18]. Nonetheless, although some reports claim that three-dimensional RFA can achieve similar results to resection for a tumor with a diameter in excess of 5 cm, most reports to date indicate the use of resection instead of RFA [4]. Regardless, the focus of the current debate is for a single liver tumor that is 3–5 cm in diameter, and for these tumors, the treatment effectiveness of RFA and resection remain to be further investigated. Thus, our study was conducted to promote an in-depth discussion on this subject.

Regarding tumor characteristics, there were more cases of the tumor located in the center of liver in the RFA group than in the resection group, mainly due jointly to preoperative CT evaluation and intraoperative examination of tumor features and liver cirrhosis. That is, when the tumor is located in the periphery of the liver, surgical resection is relatively easy, especially for a tumor in the left lateral lobe; in contrast, RFA ablation is prone to injuring surrounding tissues, such as the stomach or colon [6]. In addition, the implementation of RFA for peripheral small liver tumors may cause tumor rupture and result in metastasis [4, 19]. When the tumor is located in the center of the liver, however, especially at the junction of the donor in segments V, VI, VII, and VIII of the right side of the liver, resection will result in the loss of a large amount of normal liver tissue, leaving too small a volume in the residual liver [19]. Because much of the liver is cirrhotic in these patients, postoperative liver function cannot satisfy organ metabolism, leading to liver failure or even death. Moreover, when the tumor is close to large blood vessels, the result of RFA is poor, and part of the tumor tissue often remains. Therefore, in the clinical application of RFA, we need to consider not only the diameter of the tumor but also its location, the surrounding tissue and the underlying liver condition to achieve the best results.

The small incision with RFA surgery tends to result in significantly less blood loss; cases requiring blood transfusion were rare in this study because the blood loss during resection surgery was also small in our hospital. Therefore, although blood loss differed between the two groups, there was no significant difference in the rate of blood transfusion. Because the trauma of resection is relatively great, the surgery requires partial occlusion. The most commonly used method is semi-liver occlusion, which can prevent injury to the remaining liver due to continuous occlusion by ischemia-reperfusion. In our analysis, the intraoperative time was short, the blood loss during surgery was small, and the postoperative hospital stay was short; however, there was still a significant difference between the total treatment expense for the RFA and resection groups, mainly because domestic hospitals generally use imported RFA needles. The RFA needle costs nearly 10,000 RMB Yuan, which accounts for most of the treatment cost of RFA, whereas the overall expense of surgical resection is low. Hence, there was no difference in total treatment expense between the groups. Through the observation of postoperative complications, we found that although the occurrence rates of postoperative complications and serious complications were higher among the patients in the resection group than in the RFA group, this difference was not statistically significant. One possible reason may be that our sample size was not large enough and that abdominal surgery was performed for all our cases of RFA. Therefore, in comparison with other statistical analyses, our data are more objective and accurate. Regardless, a multi-center randomized comparison and a large sample are needed to further explore the occurrence of postoperative complications for the two methods.

Our analysis indicated similar postoperative 1-, 3-, and 5-year survival rates for the RFA and resection groups, which is similar to the results of the 18th national statistical analysis of Japan in which a statistical analysis of over 10,000 cases of liver cancer with level A liver function was conducted. In that analysis, RFA not only achieved a similar result to resection for liver tumors smaller than 2 cm but also for liver tumors 2–5 cm in diameter, with the observations extending up to 10 years [20]. Our univariate and multivariate analyses of factors contributing to overall survival and tumor-free survival rates indicated that neither resection nor RFA influenced overall survival or tumor-free survival. Our study again corroborates this point.

There are some limitations of this study. Although the sample size was relatively large, all patients were from a single center, and the study of patients from multiple centers is more persuasive. In addition, this was a retrospective analysis: we retrospectively collected and compared the characteristics of two groups of patients. Because our selection of resection or RFA before and during surgery is mainly determined according to the position of the tumor on preoperative CT and during the surgery, the method was not assigned randomly. Hence, a multi-center randomized comparative study with a large sample will be more persuasive, and this is the direction of our future work.

## Conclusion

Because there are fewer complications after RFA surgery, which has better intraoperative and postoperative performance and a postoperative survival rate comparable to that of resection surgery, abdominal RFA can be considered for wide application to single tumors with diameters of 3–5 cm, especially for cases in which the tumor is in a central location.

## Data Availability

All of the raw data and materials can be obtained by sending an E-mail to the corresponding author (ljydoctor11@163.com).

## Abbreviations

AFP: Alpha-fetoprotein
BMI: Body mass index
F: Female
HBV: Hepatitis B virus
HCC: Hepatocellular carcinoma
HIFU: High-intensity focused ultrasound
ICU: Intensive care unit
LT: Liver transplantation
M: Man
MELD: Model for end-stage liver disease
NLR: Neutrophil-lymphocyte ratio
RFA: Radiofrequency ablation
